# Identification of BELL Transcription Factors Involved in Nodule Initiation and Development in the Legumes *Pisum sativum* and *Medicago truncatula*

**DOI:** 10.3390/plants9121808

**Published:** 2020-12-20

**Authors:** Alexandra V. Dolgikh, Elizaveta S. Rudaya, Elena A. Dolgikh

**Affiliations:** All-Russia Research Institute for Agricultural Microbiology, Podbelsky chausse 3, Pushkin, 196608 St. Petersburg, Russia; sqshadol@gmail.com (A.V.D.); rudaya.s.e@gmail.com (E.S.R.)

**Keywords:** *Pisum sativum*, *Medicago truncatula*, BELL transcription factors, genomic and transcriptomic data, phylogenetic tree, gene expression, two hybrid yeast system

## Abstract

Single three-amino acid loop extension (TALE) homeodomain proteins, including the KNOTTED-like (KNOX) and BEL-like (BELL) families in plants, usually work as heterodimeric transcription factor complexes to regulate different developmental processes, often via effects on phytohormonal pathways. Nitrogen-fixing nodule formation in legumes is regulated by different families of homeodomain transcription factors. Whereas the role of KNOX transcription factors in the control of symbiosis was studied early, BELL transcription factors have received less attention. Here, we report the identification and expression analysis of *BELL* genes in the legume plants *Medicago truncatula* and *Pisum sativum*, which are involved in regulating symbiosis initiation and development. A more precise analysis was performed for the most significantly upregulated *PsBELL1-2* gene in pea. We found that the PsBELL1-2 transcription factor could be a potential partner of PsKNOX9. In addition, we showed that PsBELL1-2 can interact with the PsDELLA1 (LA) protein-regulator of the gibberellin pathway, which has a previously demonstrated important role in symbiosis development.

## 1. Introduction

The formation of symbiotic nitrogen-fixing nodules represents a process in which the development of new organs substantially depends on external factors [1,2]. In plants, such regulation can be achieved by means of a complex network of transcription factors and phytohormones. However, the exact mechanisms underlying such interactions to regulate nodulation remain unknown.

Three-amino acid loop extension (TALE) homeodomain transcription factors play a comprehensive role in the regulation of numerous biological processes, and members of this superfamily are highly conserved in different organisms [3]. In plants, two families of this superfamily have been discovered, KNOX and BELL. The significant role of TALE proteins in meristem maintenance and morphogenesis regulation has been shown, but specific processes such as chloroplast development, cell wall modification, or leaf formation also represent examples of TALE-regulated processes in plants [4,5,6].

The special feature of KNOX and BELL transcription factors is their ability to control the expression of genes involved in the regulation of biosynthesis and the degradation of phytohormones, in particular, cytokinins and gibberellins. For example, it has been shown that in the shoot apical meristem, the KNOX transcription factor SHOOT-MERISTEMLESS (STM) in *Arabidopsis thaliana* can affect the expression of *GA20ox* and *GA2ox* genes, which are responsible for the biosynthesis of active forms of gibberellin and their degradation, respectively [7]. It was also found that StBEL5 from *Solanum tuberosum* forms a heterodimeric complex with StPOTH1 (KNOX family), which is extremely important for the regulation of *GA20ox* expression through the direct binding of this complex to the promoter of the *GA20ox* gene [8]. It is important to note that KNOX and BELL also have important features such as the ability to move from cell to cell. It has been shown that such movement is achieved through plasmodesmata, and this trafficking is specific [9]. For BELL transcription factors, the long-distance transport ability with phloem flow has been observed wherein it could move from the shoot to the root.

In addition to participating in shoot developmental processes, KNOX and BELL transcription factors also play an important role in root development. It has been shown that members of the KNOX family, such as KNAT6 and BP (KNAT1), influence the development of lateral roots in *Arabidopsis*, and BLH6 (BELL family) is also involved in this regulation [8,10,11]. The role of KNOX transcription factors that belong to class II in the control of new root organ development, specifically the nitrogen-fixing nodules, has been previously examined in several studies. It was demonstrated that the expression of *KNOX* class II genes is associated with the early stages of nodule development in *Medicago truncatula* and *Pisum sativum* L. [12]. The role of *MtKNOX3*/*PsKNOX3* genes in this process has been studied in detail [13,14]. It has been found that the KNOX3 transcription factor might influence the expression of cytokinin biosynthesis genes such as *IPT*s and *LOG*s during nodulation [13,14].

However, despite the importance of the BELL–KNOX heterodimer complex formation for its functional activity, no data have been presented about the role of BEL-like transcription factors in the control of nodule development. In addition, in contrast to the well-studied mechanism of KNOX and BELL with respect to the effect on gibberellin and cytokinin metabolism in shoots, few data are available about the influence of TALE on phytohormonal processes in roots and nodules. Nevertheless, it has been shown that the exogenous treatment of *S. tuberosum* roots with gibberellins (GA3) is associated with decreased expression of several *KNOX* genes [9]. Moreover, it was recently shown that the expression levels of several *KNOX* and *BELL* genes are essentially decreased in *P. sativum* mutants, *la cry-s* (*della1 della2*), with enhanced sensitivity to gibberellins due to impaired DELLA protein (negative regulators of plant response to these hormones) function [15].

To verify the role of BELL transcription factors in the regulation of symbiosis development in legumes, a genome-wide search of *BELL* genes was performed in the model legume *M. truncatula* and crop legume pea (*P. sativum*), and phylogenetic relationships between these genes in legumes and in *Arabidopsis thaliana* and *Oryza sativa* were determined. An analysis of transcriptomic data allowed for the identification of *BELL* genes showing activated expression upon nodulation in *M. truncatula*. The expression levels of close homologues of these genes in pea were further evaluated using RT-qPCR analysis. This allowed for a more precise analysis of distinct *BELL* genes activated during symbiosis development, performed in pea.

## 2. Results

### 2.1. Diversity of BEL-Like Genes in Legumes

**Figure 1:**
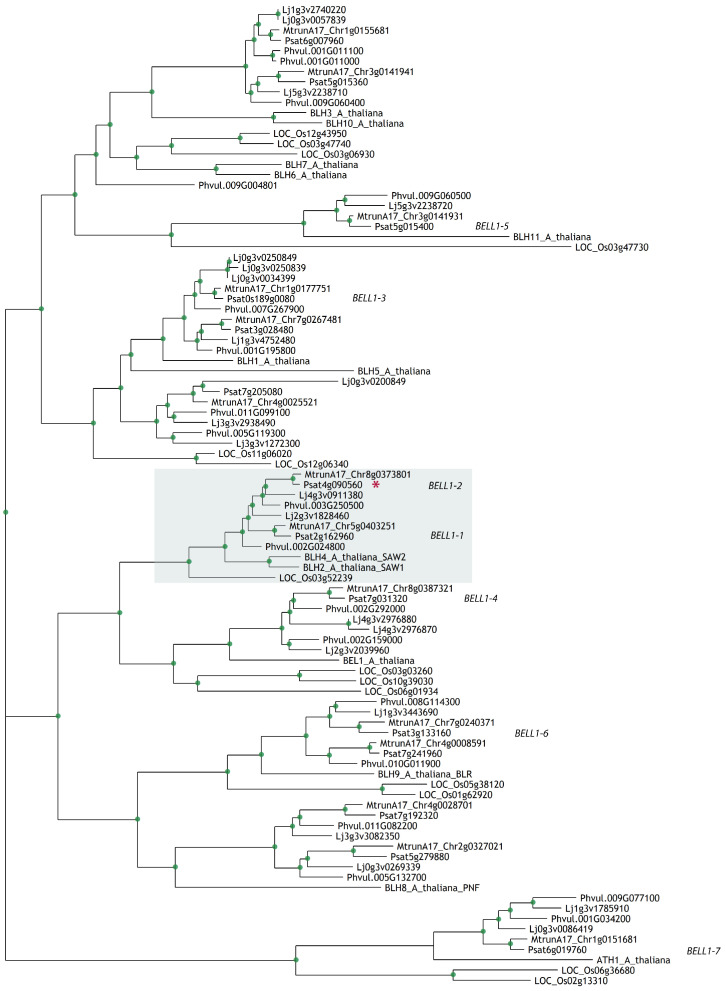
Phylogenetic tree constructed by the maximum-likelihood method based on amino acid sequences of BELLs from *Arabidopsis thaliana*, rice *Oryza sativa* and legumes: *Medicago truncatula, Pisum sativum, Lotus japonicus, and Phaseolus vulgaris*. Green dots indicate support more than 70 based on 1000 bootstrap replicates. Red asterisk indicates the gene of interest.

To identify *BELL* genes in the genome of the model legume *M. truncatula* and the crop legume pea *P. sativum*, we searched for these genes using amino acid sequences of *BEL1* and *BEL*-like genes of *A. thaliana* for BLASTP analysis [16]. As a result, we found 14 genes in the *M. truncatula* genome sequence v5 [17] and in *P. sativum* genome sequence v1 [18]. A phylogenetic tree was constructed to assess the homology of the identified genes with *A. thaliana BELL* genes. We found out that only four *Arabidopsis* genes (*AtBEL1*, *AtBLH5*, *AtBLH11*, and *AtATH1*) have unique orthologs in *P. sativum* and *M. truncatula* genomes (Figure 1). Based on this tree reconstruction, we determined the phylogenetic relationships among *BELL* genes in the legumes *M. truncatula*, *P. sativum*, *Lotus japonicus, Phaseolus vulgaris*, and non-legumes *A. thaliana, O. sativa* (Table S1).

Most of the available transcriptomic data (RNA-seq) for early stages of symbiosis development in *M. truncatula* were analyzed using the v4 genome sequence as a reference. Since our analysis allowed us to identify 14 *BELL* genes in *M. truncatula* genome v5 and only 13 *BELL* genes were found in *M. truncatula* genome v4, we performed re-analysis of these available transcriptomic data (RNA-seq) using *M. truncatula* genome sequence v5 as a reference. Data collected at 24 and 48 h and 3, 4, 5, and 7 days after inoculation (3, 4, 5, and 7 dai) were used for analysis (Figure S1). We found that the expression level of some *BELL* genes had a similar level in the non-inoculated and inoculated roots; however, for other *BELL* genes their expression level was significantly increased in response to inoculation (*MtBELL1-2*, *MtBELL1-3*, and *MtBELL1-4*) (MtrunA17_Chr8g0373801, MtrunA17_Chr1g0177751, and MtrunA17_Chr8g0387321, correspondently). For *MtBELL1-5* (MtrunA17_Chr3g0141931), strong upregulation (up to 10-fold change) has been shown, although its expression level remained relatively low in inoculated roots.

**Figure 2:**
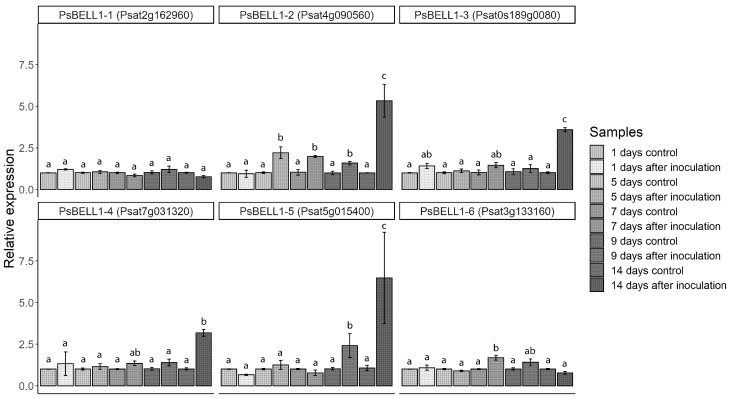
Expression dynamics of selected *BELL* genes in inoculated *Pisum sativum* roots (cv. Frisson) at different stages of symbiosis development. mRNA levels were normalized against *Ubiquitin* and values were calculated as ratios relative to non-inoculated root expression levels (control). The data of three independent biological experiments were analyzed. For one biological experiment, three technical replicates were obtained at each developmental stage. Bars represent the mean ± SEM. Different letters indicate statistically significant differences between groups as analyzed by one-way analysis of variance (one-way ANOVA), followed by Tukey’s post hoc test.

To evaluate the expression level of homologous *BELL* genes in *P. sativum* roots after inoculation, we performed RT-qPCR analysis (Figure 2, Figure S2). The expression dynamics of *BELL* genes were evaluated at different stages of symbiosis development after inoculation of pea *P. sativum* with *Rhizobium leguminosarum bv. viciae*. We found that the expression levels of *PsBELL1-2* started to increase at the first stages of symbiosis initiation (5 days after inoculation, 5 dai) and reached the maximum level in the nodules (14 dai), as was also shown for homologue of this gene in *M. truncatula* based on transcriptomic analysis (Figure S1). In addition, we detected increased expression of *PsBELL1-3* and *PsBELL1-4* in pea roots at 7 dai and the highest level of their expression was found in the nodules.

**Figure 3:**
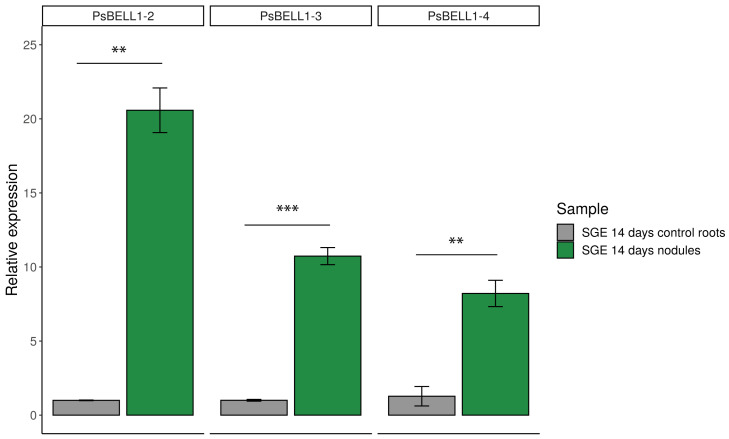
RT-qPCR analysis of *PsBELL1-2* (Psat4g090560)*, PsBELL1-3* (Psat0s189g0080) and *PsBELL1-4* (Psat7g031320) expression in pea nodules (SGE line). mRNA levels were normalized against *Ubiquitin* and values were calculated as ratios relative to non-inoculated root expression levels (control). The data of three independent biological experiments were analyzed. Bars represent the mean ± SEM. The asterisks indicate a significant induction in nodules relative to control roots based on Student’s t-test analysis (** *p* < 0.01, *** *p* < 0.001).

We also found that the expression levels of *PsBELL1-2*, *PsBELL1-3*, and *PsBELL1-4* were highly increased in the nodules of the other pea line (SGE) (Figure 3). Analysis of these gene expressions in pea nodules based on available RNA-seq data showed similar results (Figure S3). Moreover, these data corresponded to transcriptomic data in *M. truncatula* nodules [17], where homologues of these genes, *MtBELL1-2*, *MtBELL1-3*, and *MtBELL1-4*, also showed the strongest expression level in the nodules (Figures S4 and S5). Therefore, the expression of *MtBELL1-2*, *MtBELL1-3*, and *MtBELL1-4* and their putative orthologues *PsBELL1-2*, *PsBELL1-3*, and *PsBELL1-4* in pea has been shown to be upregulated during nodulation in both legumes, suggesting their possible participation in the regulation of this process.

### 2.2. Search for BELL Genes Regulated by NIN Transcription Factor during Symbiosis Development

**Figure 4:**
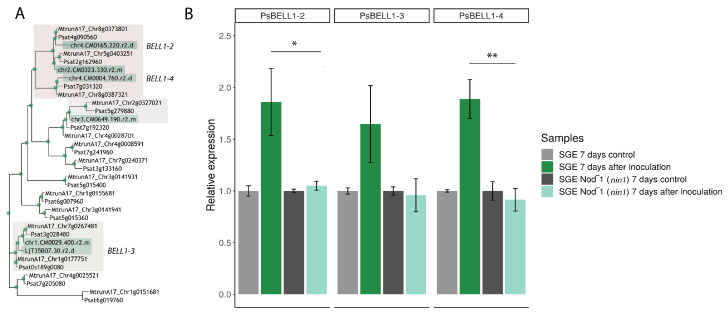
Phylogenetic tree constructed by the maximum-likelihood method based on amino acid sequences of *Medicago truncatula* and *Pisum sativum BELL* genes and *Lotus japonicus* homologous *BELL* genes detected in ChIP-Seq analysis. Green dots indicate support more than 70 based on 1000 bootstrap replicates (**A**). Expression pattern of *PsBELL1-2* (Psat4g090560)*, PsBELL1-3* (Psat0s189g0080) and *PsBELL1-4* (Psat7g031320) genes in the roots of pea SGENod^−^-1 (*Psnin-1*) mutant and wild type *P. sativum* cv. SGE (**B**). mRNA levels were normalized against *Ubiquitin* and values were calculated as ratios relative to non-inoculated root expression levels (control). The data of three independent biological experiments were analyzed. Bars represent the mean ± SEM. The asterisks indicate statistically significant differences based on one-way analysis of variance (one-way ANOVA), followed by Tukey’s post-hoc test (* *p* < 0.05; ** *p* < 0.01).

Based on data from ChIP-Seq analysis for model legume *Lotus japonicus*, the *BELL* gene (*Lj4g3v2976870.1*, *L. japonicus* genome sequence v3) was detected as a possible target of the NIN transcription factor [19]. The homologs of this gene in *M. truncatula* and *P. sativum* were determined to be *MtBELL1-4* and *PsBELL1-4*, respectively. Since the NIN transcription factor plays a central role in nodule organogenesis and infection processes, the search for additional *BELL* genes that are regulated by this protein is of great interest. Here, from ChIP-Seq in *L. japonicus*, we extracted sequences of all available *BELL* genes detected as possible targets for the NIN transcription factor. BLAST analysis for screening of *L. japonicus* ChIP-Seq was performed using *M. truncatula* and *P. sativum BELL* gene sequences as a local database. As a result, we detected five additional *BELL* genes as possible targets of the NIN transcription factor in *L. japonicus*. To establish the relationship among these genes in different legumes, we constructed a phylogenetic tree (Figure 4A). Interestingly, four of the six detected genes were identified as homologs of genes that are highly expressed in *M. truncatula*- and *P. sativum*-inoculated roots and nodules (Figures S1–S4). The homologues of *BELL1-2* and *BELL1-4* genes, which increased in expression at the early stages of symbiosis development, also had the highest enrichment score, which might indicate their regulation by the NIN transcription factor at early stages of symbiosis development (Table S2).

### 2.3. Expression of PsBELL1-2, PsBEL L1-3, and PsBELL1-4 Genes in Pea SGENod^−^-1 (Psnin-1) Mutant

To evaluate the dependence of *BELL* gene expression on the NIN transcription factor in pea, a comparative analysis was performed between wild-type pea plants (SGE line) and the pea SGENod^−^-1 (*Psnin-1*) mutant. Whereas the expression dynamics data revealed that *PsBELL1-2*, *PsBELL1-3*, and *PsBELL1-4* levels started to increase significantly at 7 dai (Figure 2), we examined whether such induction takes place in *P. sativum Psnin-1* mutant at the same stage. Indeed, we did not observe an expression increase in these genes in the SGENod^−^-1 (*Psnin-1*) mutant, in contrast to the increased expression of *PsBELL1-2*, *PsBELL1-3*, and *PsBELL1-4* in response to inoculation in the wild-type (Figure 4B). These data suggest that *PsBELL1-2*, *PsBELL1-3*, and *PsBELL1-4* are possible targets of the NIN transcription factor in pea.

### 2.4. Analysis of Interaction between PsBELL and PsKNOX Regulators during Nodulation

**Figure 5:**
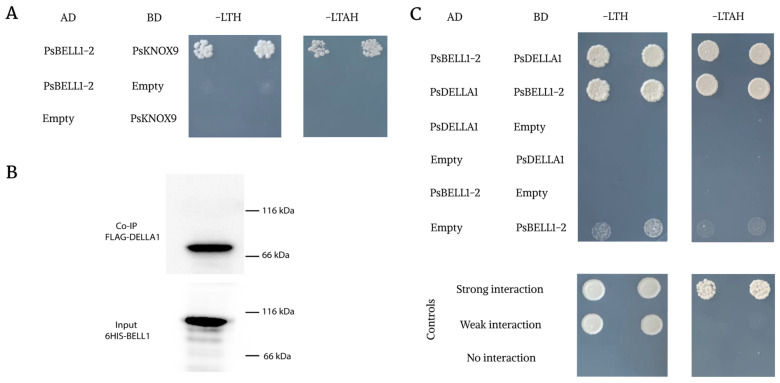
Analysis of the interaction between PsBELL1-2, PsKNOX9 and PsBELL1-2, PsDELLA1 using different approaches. Yeast two-hybrid assays between PsBELL1-2 and PsKNOX9 (**A**), PsBELL1-2 and PsDELLA1 (**C**). Co-immunoprecipitation (Co-IP) between PsDELLA1-FLAG and PsBELL1-2 (**B**). For the yeast two-hybrid assays, serial dilutions optical density at 600 nm (OD600) = 0.1 and 0.05 were used. Interaction was tested on a selective medium lacking leucine, tryptophan, and histidine (SD-LTH) and medium additionally lacking adenine (SC-LTAH). Yeast growth on indicated mediums shows the protein interaction. As controls, a few pairs of vectors (pEXP32/Krev1 and pEXP22/RalGDS-wild type, pEXP22/RalGDS-m1, and pEXP22/RalGDS-m2) suggested by the manufacturer (Thermo Fisher Scientific, Waltham, MA, USA) were used for strong, weak, and not detectable interactions.

Since it has been previously shown that BELL transcription factors might interact with KNOX transcription factors during the regulation of plant developmental processes [4], we suggested that BELL transcription factors could form complexes with KNOX regulators during symbiosis development. This might also be related to the regulation of some phytohormonal pathways. The significant induction of *MtKNOX3/PsKNOX3*, *MtKNOX5/PsKNOX5*, and *MtKNOX9/PsKNOX9* genes was found upon nodulation in these legumes. To verify this possible interaction, we performed a more precise analysis using the two hybrid yeast system (Y2H) (Figure 5A,C). The analysis was performed for PsBELL1-2 and PsKNOX3 with PsKNOX5 and PsKNOX9 transcription factors from pea, for which their activation during symbiosis development has been previously shown [12]. As a result, we were able to detect the interaction between PsBELL1-2 and PsKNOX9 in our experiments (Figure 5). Since both regulators are also activated at the early stages of symbiosis, the possibility of complex formation between such transcription factors points towards their involvement in the regulation of similar processes at these stages.

Co-immunoprecipitation analysis was performed for PsBELL1-2 tagged with 6HIS and PsDELLA1 tagged with FLAG (Figure 5B). Proteins were synthesized in *E. coli*. After extraction and incubation of the proteins on ice for 1 h, proteins were subjected to immunoprecipitation with anti-HIS resin and then analyzed on an immunoblot with anti-FLAG and anti-HIS.

### 2.5. Analysis of Interaction between the Most Important Gibberellin Network Regulators, DELLA Proteins, and BELL Transcription Factors

Since significant changes in gibberellin and cytokinin metabolism occur at the initial stages of nodulation, we studied the possible interaction between BELL transcription factors and regulators of these phytohormonal pathways. One of the most important gibberellin network regulators comprises DELLA proteins. In our previous study, we showed that PsDELLA1 and PsDELLA2 proteins had the most significant impact on pea–rhizobial symbiosis development [15]. Moreover, the expression levels of several *BELL* and *KNOX* genes were essentially decreased in the *P. sativum* mutants *la cry-s* (*della1 della2*). Here, we attempted to estimate the possible interaction between DELLA proteins and transcription factors from the BELL and KNOX families. Considering that the level of *BELL1-2* expression was the most significant during symbiosis, we performed a more detailed analysis of PsBELL1-2 and PsDELLA1 interactions using the Y2H system, as well as a co-immunoprecipitation assay. Our results reveal that PsBELL1-2 interacts with the PsDELLA1 protein based on the Y2H (Figure 5C). No interaction was observed with the empty vectors. At the same time, the co-immunoprecipitation assay to verify the PsBELL1-2 and PsDELLA1 interaction was performed (Figure 4B). Using this approach, we also confirmed the possibility of such an interaction.

## 3. Discussion

During nodule development, a complex network of transcription factors and phytohormones is involved in the regulation of the shape of this organ and its position on the root. However, this requires future investigations of the regulatory links between phytohormones and transcription factors, which are tightly interconnected in these processes. In this relationship, the contribution of components that are susceptible to phytohormonal action or can influence downstream components of phytohormonal pathways is crucial. Therefore, TALE proteins might be important regulators of nodule development.

It is well known that transcription factors of the TALE superfamily are important participants in processes associated with meristem maintenance and organ development [3,4]. However, specific members of this superfamily, which are involved in the regulation of definite processes, remain unknown. The role of KNOX proteins in the regulation of legume–rhizobial symbiosis was previously studied in detail. Several members of the KNOX family that belong to class II were found to be activated at the initial stages of nodule development. Therefore, it has been shown that *MtKNOX3/PsKNOX3*, *MtKNOX5/PsKNOX5*, *MtKNOX9/PsKNOX9*, and *MtKNOX10/PsKNOX10* play an important role in nodule organogenesis and are expressed in the nodule primordium/meristem [12,13]. However, despite the fact that KNOX and BELL function in most cases as heterodimeric complexes, no data have been reported about BELLs, which are involved in this process.

Our data showed that several members of the BELL family might be involved in the control of legume–rhizobial symbiosis development. The initial stages of symbiosis are apparently associated with the activation of some *BELL* genes. Among them, *PsBELL1-2*, *PsBELL1-3*, and *PsBELL1-4* exhibited increased expression levels in comparison with those in non-inoculated roots at the initial stages of symbiosis, as well as high expression in the nodules. Similarly, *MtBELL1-2* and *MtBELL1-4* homologous genes showed activation at the early symbiotic stages (1–2 dai) in *M. truncatula*, whereas their expression level was most significant in the nodules. In addition, *MtBELL1-3* expression was high in *M. truncatula* nodules. This suggests a possible role of these genes in the regulation of nodulation.

Among these genes, *PsBELL1-2* showed the most significant upregulation and will be used for future analysis in pea. The PsBELL1-2 transcription factor was shown to interact with the PsKNOX9 transcription factor, which suggests their participation in the regulation of common processes. Considering our data on the interaction between PsBELL1-2 and PsKNOX9, we suggest that BELL1-2 is of great interest for future studies. It has recently been shown that the closest homologues of PsBELL1-2 in *A. thaliana* (AtBLH2/AtBLH4) can influence the expression of genes encoding pectin methyl transferases (PMEs), which are important components of plant cell wall modification and are involved in the demethylesterification of homogalacturonan [20]. Interestingly, it has recently been shown that the decline of methyl esterified homogalacturonan content is specifically associated with infection thread development during nodulation [21]. Some pea mutants, SGEFix^−^-5 (*sym33-2/ipd3*) [22] and SGEFix^−^-2 (*sym33-3/ipd3*) [23], have impaired infection processes (blocking rhizobia release from infection threads) and form noneffective nodules. Previously, we found significantly decreased levels of *BELL1-2* in the nodules of these mutants. It would thus be interesting to analyze the link between PsBELL1-2 activation and the level of *PME* gene induction in nodules of the *sym33/ipd3* mutants.

It is important to note that several *BELL* genes were found in the ChIP-seq analysis of legume plants as possible targets of the NIN transcription factor, which is one of the most important regulators of nodule organogenesis and infection. A phylogenetic tree revealed that *BELL*s with the highest enrichment score also showed the highest increase in their expression level in nodules. This suggests that the expression of these *BELL* genes during symbiosis development might be dependent on NIN. Since the expression levels of several *BELL* genes are quite high in roots, temporal and spatial regulation of the expression of individual *BELL* genes by the NIN transcription factor might be important for their selective activation during nodulation. In contrast, only one *KNOX* gene was detected in the ChIP-Seq analysis of legume plants (its closest homolog in pea is *PsKNOX9*). Since we found that the expression dynamics of *PsBELL1-2* and *PsKNOX9* genes are highly similar, the possibility of their interaction is consistent with data suggesting that both of them could be regulated by NIN. However, the involvement of other KNOX regulators in the process of symbiosis development remains unclear.

Since it was shown that BELLs are involved in the regulation of genes associated with gibberellic acid metabolism, the ability of PsBELL1-2 to interact with such components of the gibberellin signaling pathway, as is the case for PsDELLA1, is of great interest. It was shown that DELLA proteins are extremely sensitive to the presence of active gibberellins and are quickly degraded via the SCF-E3 complex in the presence of gibberellins in the environment. Considering the ability of *BELL* and *KNOX* genes to influence phytohormonal metabolism, such interactions might explain the mechanism by which TALE proteins influence gibberellin content. It is interesting to note that recent data showed the interaction between DELLA proteins and the closest homologues of PsBELL1-2 in the model plant *A. thaliana*, specifically AtBLH2/AtBLH4 [24]. This suggests a common tendency in the regulation of BELL transcription factors through their interaction with DELLA proteins.

## 4. Materials and Methods

### 4.1. Phylogenetic Reconstruction

A phylogenetic tree was constructed using the maximum-likelihood method with the IQ-TREE tool v.1.6.1 [25], based on amino acid sequences that were aligned using the PRANK tool v.170427 with default settings [26]. The bootstrap values were obtained from 1000 bootstrap replicates using ultrafast bootstrap [27]. *Arabidopsis thaliana* amino acid sequences were retrieved from the UniProt database using previously published accession numbers [3]. For the identification of *Arabidopsis BELL* homologues in *P. sativum* v1 and *M. truncatula* v5 genomes, the BLASTP tool v.2.6.0 (word size = 3) [16] was used with an E-value threshold of 10^−30^. In the phylogenetic tree for identification of *L. japonicus* genes from ChIP-Seq [19], we used BLASTP to detect *BELL* genes among the enriched genes, and the same pipeline for tree construction was used for this analysis. The R package ggtree was used for visualization in both cases [28]. All the sequences of *BELL* genes for *A. thaliana, O. sativa*, and the legumes can be found in the Supplementary Materials.

### 4.2. RNA-Seq Quantification and Statistical Analysis

Raw reads from the RNA-Seq project PRJNA552042 [29], including data for 24 and 48 h and 3, 4, 5, and 7 days after inoculation, and mock treatment data for the same stages, were used for analysis. The *Medicago truncatula* genome version 5.0 was used as a reference. The reads were mapped to the genome using HISAT2 [30] tool v.2.1.0 with default parameters, and the raw counts were calculated with help of the featureCounts function from the Subread package [31]. The EdgeR package was used to calculate CPM values [32].

### 4.3. Bacterial Strains and Inoculation

Inoculation of the pea plants was performed with the *Rhizobium leguminosarum* biovar *viciae* strain 3841 [33] and CIAM1026 [34]. The bacterial liquid culture was grown at 28 °C in B^−^ medium [35] for 24–48 h, diluted up to the optical density at 600 nm (OD600) 0.5 and applied to plants one day after planting.

### 4.4. Plant Material and Growth Conditions

*P. sativum* L. cv. Frisson plants were used to examine the expression dynamics of *BELL* genes. Wild type SGE line [36] and its derived mutant SGENod^−^-1 (*Psnin-1*) [37] were also used in this study to evaluate expressions of *PsBELL1-2*, *PsBELL1-3*, and *PsBELL1-4*. The SGE nodules were collected at 14 days after inoculation (14 dai) and used to assess the expression level of *BELL* genes.

Gene expression experiments were carried out using the pea seedlings collected at different stages after inoculation. Non-inoculated roots were also collected at similar developmental stages and used as a control. To grow the seedlings, the seeds were sterilized using sulfuric acid for 5 min, washed 3 times with water, transferred on 1% agar plates, and allowed to germinate at 23 °C in the dark. Then, 4–5-day plant seedlings were transferred into pots with vermiculite saturated with Jensen medium [38], grown in a growth chamber at 21 °C and 16 h light/8 h dark cycle with 60% humidity. Fragments of the main roots (responsive zone starting from 5 to 6 mm from the root tip) or fragments of main roots with primordia/nodules were collected at day 7 for the SGE and SGENod^−^-1 (*Psnin-1*) mutant. For the analysis of dynamics, the samples were collected at different stages after inoculation (1, 3, 5, 7, 9, 11, and 14 dai). Fragments of non-inoculated main roots were collected at similar developmental stages. Plant material for the gene expression studies was immediately immersed in liquid nitrogen and stored in a freezer at −80 °C.

### 4.5. RNA Extraction, cDNA Synthesis, and Quantitative Reverse Transcription PCR (RT-qPCR)

Approximately 50–100 mg of ground plant tissue was used for RNA extraction, as previously described [39]. Around 1–2.5 μg of total RNA was used to synthesize cDNA using 20 μL RevertAid Reverse Transcriptase (Thermo Fisher Scientific, USA), and then the cDNA samples were diluted to 1:10. Then, 2 µL of cDNA was used for quantitative PCR using Bio-Rad iQ Sybr master mix (Bio-Rad Laboratories, Hercules, CA, USA) following the manufacturer’s recommendations. The CFX-96 real-time PCR detection system with C1000 thermal cycler (Bio-Rad Laboratories, USA) was used for analysis. Cycle threshold (Ct) values were obtained using the accompanying software and data were analyzed according to the 2^−ΔΔCt^ method [40]. All reactions were performed in triplicate and averaged. For gene expression quantification, the used primer pairs are shown in Table S3. All primers were produced by Evrogen (Moscow, Russia). The gene expression was normalized against the constitutively expressed *Ubiquitin* gene in pea. For temporal *BELL* gene expression, each replicate contained tissue of 3–4 plants, and the experiments were repeated thrice.

### 4.6. RT-qPCR Data Analysis

One-way analysis of variance (one-way ANOVA) with post-hoc Tukey test or Student’s *t*-test and relevant R function were used to estimate the statistical significance. Bar plots were used to represent the relative expression values (2^−ΔΔCt^) ± SEM for three biological replicates.

### 4.7. Cloning of PsDELLA1, PsKNOX3, and PsBELL1 Genes for Yeast Transformation

The full-length coding sequences of *PsDELLA1*, *PsKNOX9*, and *PsBELL1* genes were obtained by amplifying the cDNA of cv. Finale using specific PCR primer pairs flanking with attB1 and attB2 sequences or CACC sequence in the forward primer (Table S3) for subsequent cloning in pDONR221 or pENTRY vectors (Thermo Fisher Scientific, USA).

Then, these sequences were finally subcloned into the destination vectors pDEST22 (PREY) or pDEST32 (BAIT) using the LR clonase enzyme (Thermo Fisher Scientific, USA). All the verified constructs were transferred into the pJ6969-4A yeast strain.

### 4.8. Yeast Two-Hybrid Assay (GAL4 Transcription Factor-Based Assay)

The *Saccharomyces cerevisiae* strain pJ6969-4A [41] was transformed simultaneously with pDEST22 and pDEST32 vectors for GAL4-based selection. To transform *S. cerevisiae* pJ6969-4A, the protocol for preparation of chemically competent cells was used [42]. A few pairs of vectors (pEXP32/Krev1 and pEXP22/RalGDS-wild type, pEXP22/RalGDS-m1, and pEXP22/RalGDS-m2) suggested by the manufacturer as controls were used for the strong, weak, and not detectable interactions. Analyses of interactions were conducted on selective media such as synthetic complete (SC)-LT (without leucine and tryptophan), SC-LTH (without leucine, tryptophan, and histidine), and SC-LTAH (without leucine, tryptophan, adenine, and histidine).

### 4.9. Protein Synthesis in E. coli and Co-Immunoprecipitation Assay

Co-immunoprecipitation was carried out using a µMACS kit (Miltenyi Biotec, Bergisch Gladbach, Germany) containing MicroBeads with immobilized anti-HIS antibodies. The coding sequences of *PsBELL1-2* and *PsDELLA1* genes were amplified on the cDNA and cloned in a pRSETa vector. The synthesis of proteins was performed in *E. coli* C41 cells. The pellets of *E. coli* cells were resuspended in a lysis buffer (50 mM tris-HCl pH 8.0, 1% Triton X-100, and 150 mM NaCl) and the cell lysates were obtained using sonication (3 × 20 s). These lysates, containing PsBELL1-2 tagged with 6HIS and PsDELLA1 tagged with FLAG proteins, were co-incubated with the MicroBeads for 1 h on ice, and then they were loaded onto a MACS column placed in the magnetic field of a µMACS separator. Next, the column with associated proteins was washed twice with the lysis buffer. After elution with a denaturing elution buffer, the precipitated proteins were analyzed using SDS-PAGE.

## Supplementary Materials

The following are available online at https://www.mdpi.com/2223-7747/9/12/1808/s1, Table S1: Relationships between IDs of *P. sativum*, *M. truncatula* and *A. thaliana BELL* genes based on phylogenetic tree reconstruction; Table S2: IDs of *L. japonicus BELL* genes detected in ChIP-Seq analysis [18] as possible targets of NIN transcription factor and relevant fold enrichment score; Table S3: List of primers; Figure S1: Expression level of *M. truncatula BELL* genes based on re-analysis of RNA-Seq data from PRJNA552042 project using *M. truncatula* genome v.5 as a reference. Figure S2: Graphic illustration of data for *P. sativum BELL* genes expression in the roots and nodules based on RNA-Seq analysis; Figure S3: Graphic illustration of data for *P. sativum BELL* gene expression in the roots and nodules based on RNA-Seq analysis [43] using *P. sativum* genome sequence v1 as reference. Figure S4: Expression level of *M. truncatula BELL* genes in roots based on re-analysis of RNA-Seq data. Figure S5: Expression level of *M. truncatula BELL* genes in the different nodule zones. Data file: sequences of *BELL* genes for *A. thaliana, O. sativa* and legumes.
