# Supplementary material for: Identification of BELL Transcription Factors Involved in Nodule Initiation and Development in the Legumes Pisum sativum and Medicago truncatula
Source: Plants (Basel). 2020 Dec 20;9(12):1808. doi: 10.3390/plants9121808 (PMC7766112; doi:10.3390/plants9121808)
Supplement: Supplementary file 1 [file plants-09-01808-s001.pdf]

Supplementary materials

Table S1. Relationships between IDs of *P. sativum*, *M. truncatula* and *A. thaliana BELL* genes based on phylogenetic tree reconstruction (Figure 1).

**MtV5 ID**	**MtV4 ID**	***Pisum sativum v1***	***Arabidopsis thaliana***	**Our names**
MtrunA17_Chr1g0151681	Medtr1g016490	Psat6g019760	ATH1	BELL1-7
MtrunA17_Chr1g0155681	Medtr1g023050	Psat6g007960	BLH3/BLH10	-
**MtrunA17_Chr1g0177751**	**Medtr1g057790**	**Psat0s189g0080**	BLH1	**BELL1-3**
MtrunA17_Chr2g0327021	Medtr2g095050	Psat5g279880	BLH8	-
MtrunA17_Chr3g0141931	Medtr3g112290	Psat5g015400	BLH11	BELL1-5
MtrunA17_Chr3g0141941	Medtr3g112300	Psat5g015360	BLH3/BLH10	-
MtrunA17_Chr4g0008591	Medtr4g019450	Psat7g241960	BLH9	-
MtrunA17_Chr4g0025521	Medtr4g051532	Psat7g205080	BLH5	-
MtrunA17_Chr4g0028701	**NA**	Psat7g192320	BLH8	-
MtrunA17_Chr5g0403251	Medtr5g018860	Psat2g162960	BLH2/BLH4	BELL1-1
MtrunA17_Chr7g0240371	Medtr7g065050	Psat3g133160	BLH9	BELL1-6
MtrunA17_Chr7g0267481	Medtr7g106320	Psat3g028480	BLH1	-
**MtrunA17_Chr8g0373801**	**Medtr8g078480**	**Psat4g090560**	BLH2/BLH4	**BELL1-2**
**MtrunA17_Chr8g0387321**	**Medtr8g098815**	**Psat7g031320**	BEL1	**BELL1-4**

Table S2. IDs of *L. japonicus BELL* genes detected in ChIP-Seq analysis [18] as possible targets of NIN transcription factor and relevant Fold Enrichment Score.

***Lotus japonicus* (v 2.5) ID**	***Lotus japonicus* (v 3.0) ID**	**Fold enrichment**	**Ortholog in *M. truncatula* (ID from v5)**	**Ortholog in *M. truncatula* (ID from v4)**	**Ortholog in *P. sativum***
chr4.CM0165.220.r2.d	Lj4g3v0911380.1	26.78	MtrunA17_Chr8g0373801	Medtr8g078480, MtBELL1-2	Psat4g090560
chr1.CM0029.400.r2.m	Lj1g3v4752480.1	23.95	MtrunA17_Chr7g0267481	Medtr7g106320	Psat3g028480
chr4.CM0004.760.r2.d	Lj4g3v2976870.1, Lj4g3v2976880.1	18.97	MtrunA17_Chr8g0387321	Medtr8g098815, MtBELL1-4	Psat7g031320
LjT35B07.30.r2.d	Lj0g3v0034399.1, Lj0g3v0250849.1	10.4	MtrunA17_Chr1g0177751	Medtr1g057790, MtBELL1-3	Psat0s189g0080
chr2.CM0323.330.r2.m	Lj2g3v1828460.1	6.21	MtrunA17_Chr5g0403251	Medtr5g018860	Psat2g162960
chr3.CM0649.190.r2.m	Lj3g3v3082350.1	3.17	MtrunA17_Chr2g0327021	Medtr2g095050	Psat5g279880

A.

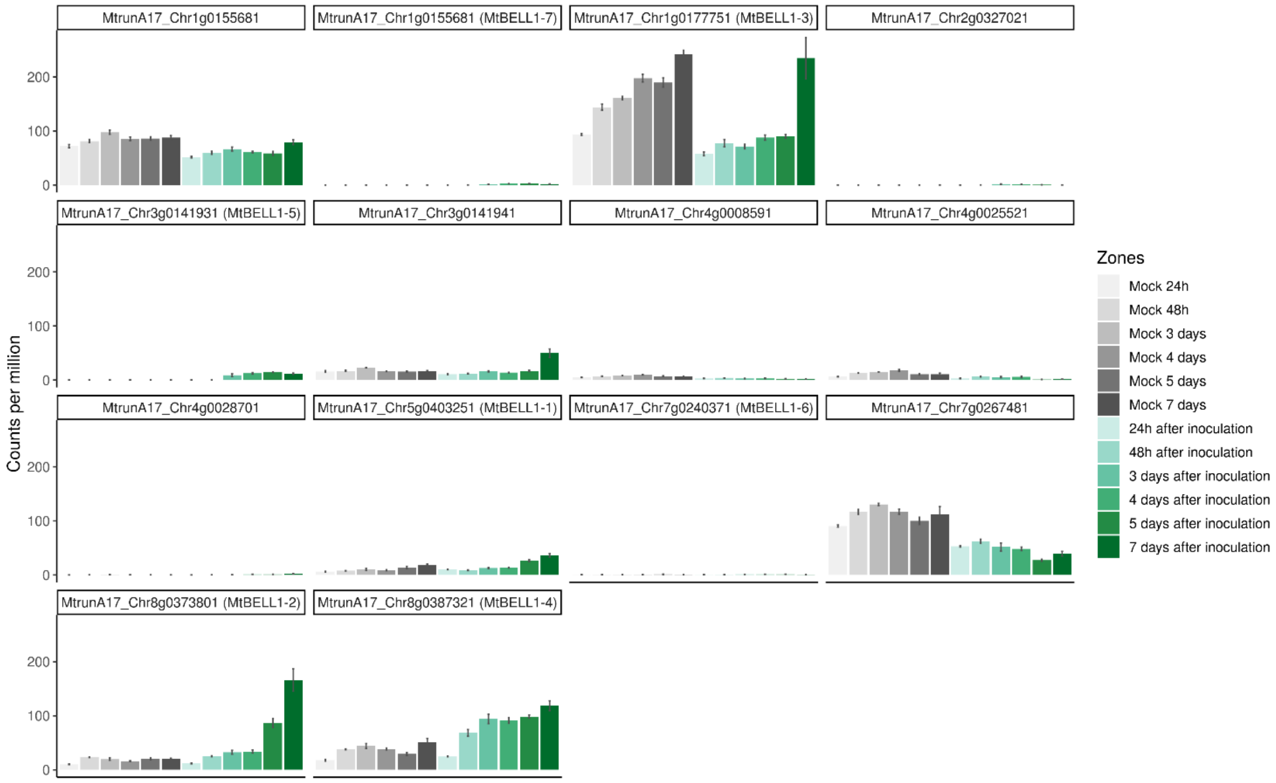

B.

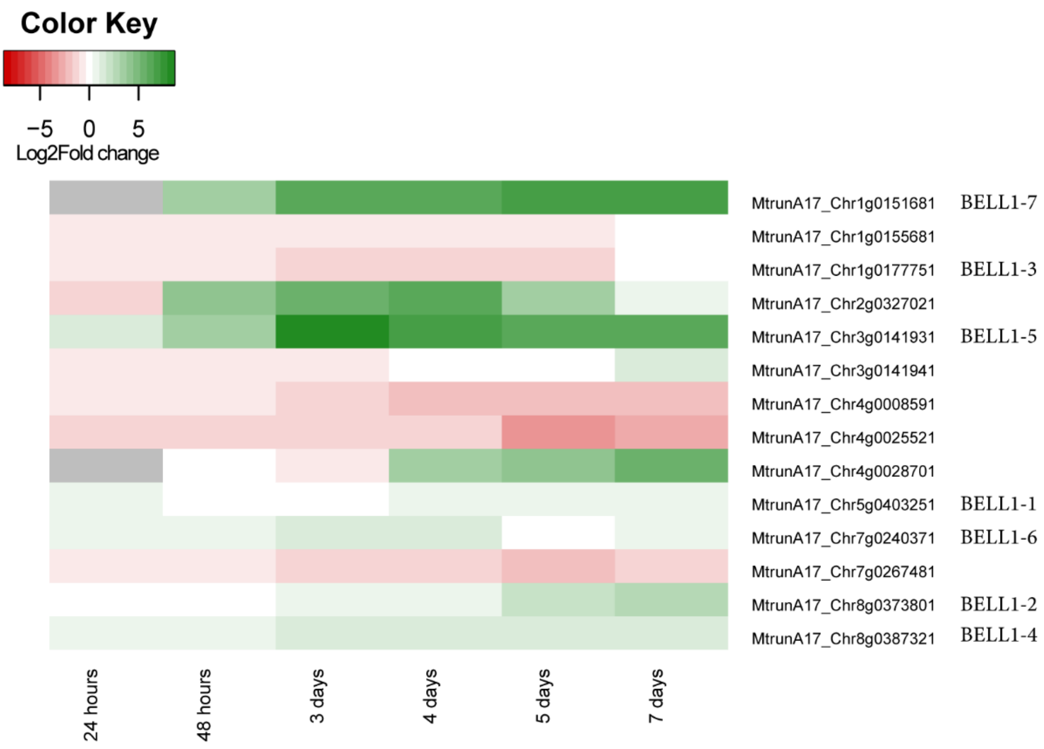

Figure S1. A. Expression level of *M. truncatula BELL* genes based on re-analysis of RNA-Seq data from PRJNA552042 project [27] using *M. truncatula* genome v.5 as a reference. Bars represent means of counts per million values (CPM) based on 4-6 replicates. B. Heatmap of the *M. truncatula BELL* gene expression values as log2fold changes at 24 and 48 h and 3, 4, 5, and 7 days after inoculation.

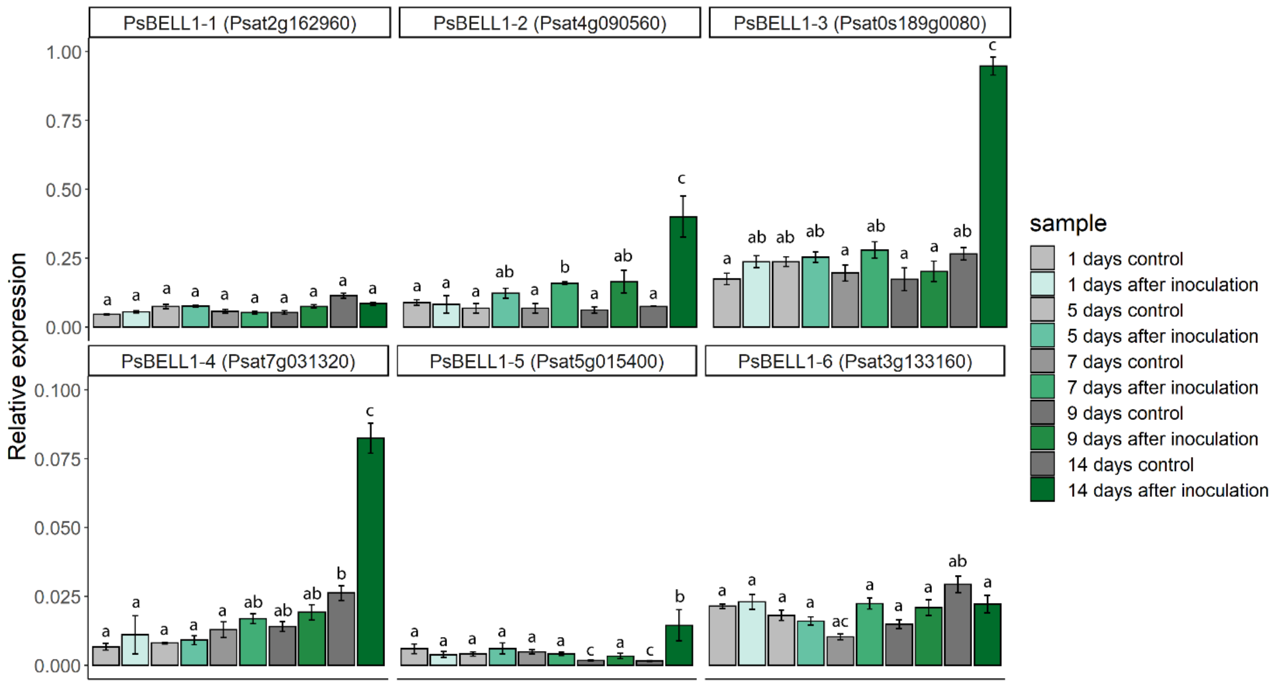

Figure S2. Expression of *PsBELL* genes normalized to the reference *Ubiquitin* gene. The data of three independent biological experiments were analyzed. For one biological experiment three technical replicates were obtained at each developmental stage. Bars represent the mean of raw expression values ($2^{-deltaCt}$) normalized to the maximum value between all genes ± SEM. Different letters indicate statistically significant differences between groups as analyzed by One-way analysis of variance (One-way ANOVA), followed by Tukey's post hoc test.

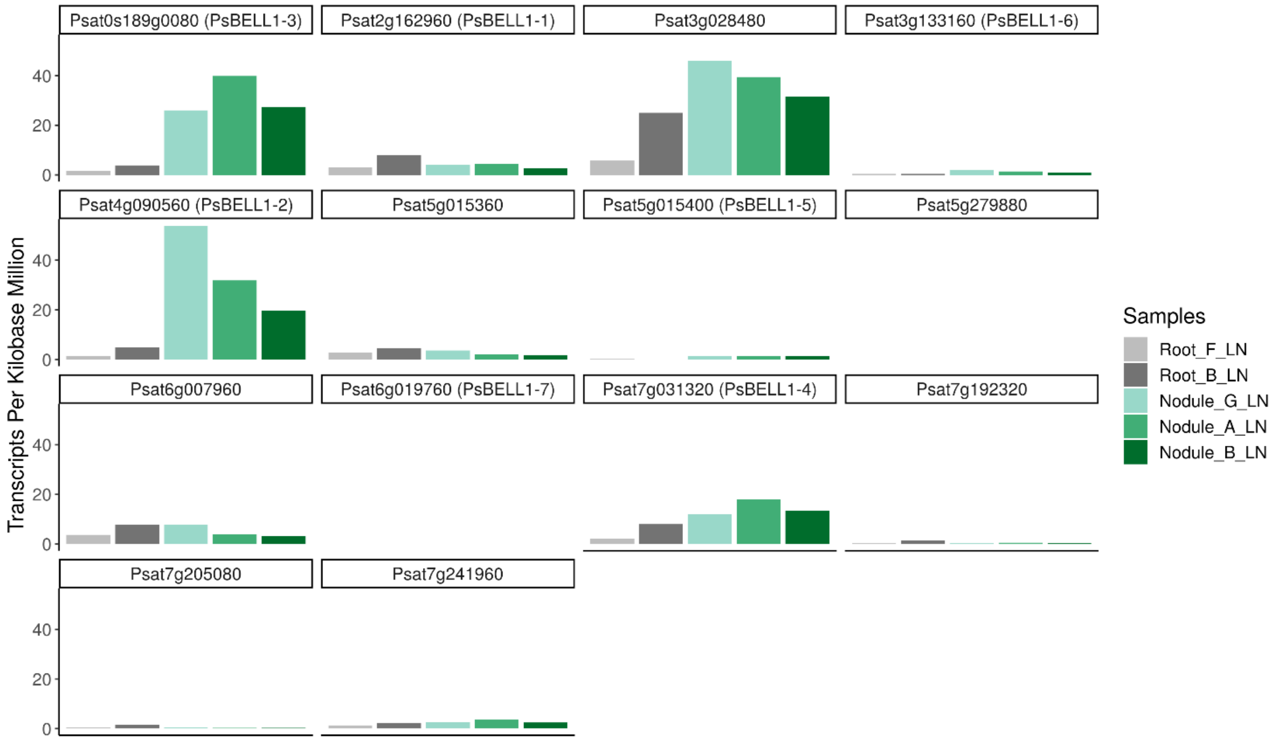

Figure S3. Graphic illustration of data for *P. sativum BELL* gene expression in the roots and nodules (cv. Cameor) based on RNA-Seq analysis [43] using *P. sativum* genome sequence v1 as reference. Roots: F - 8 days after sowing; B - start of flowering. Nodules: G - 18 days after sowing, i.e. 10 days after inoculation; A - 7–8 nodes, 5–6 opened leaves; B - start of flowering. Columns represent means of Transcripts Per Kilobase Million values (TPM).

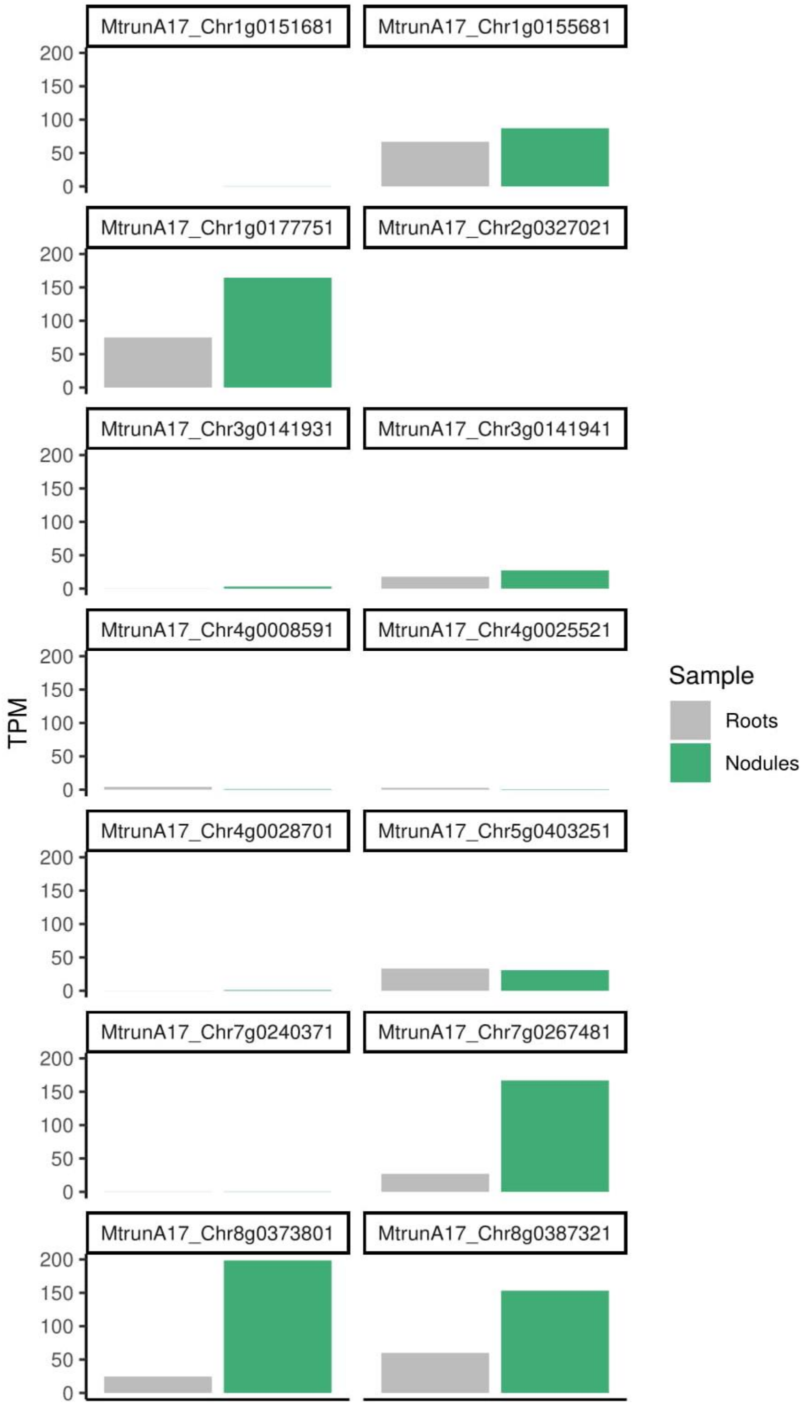

Figure S4. Expression level of *M. truncatula BELL* genes in the nodules and roots based on re-analysis of RNA-Seq data [16]. Bars represent means of TPM values for 3 replicates. MtrunA17_Chr8g0373801 (*MtBELL1-2*), MtrunA17_Chr1g0177751 (*MtBELL1-3*), and MtrunA17_Chr8g0387321 (*MtBELL1-4*).

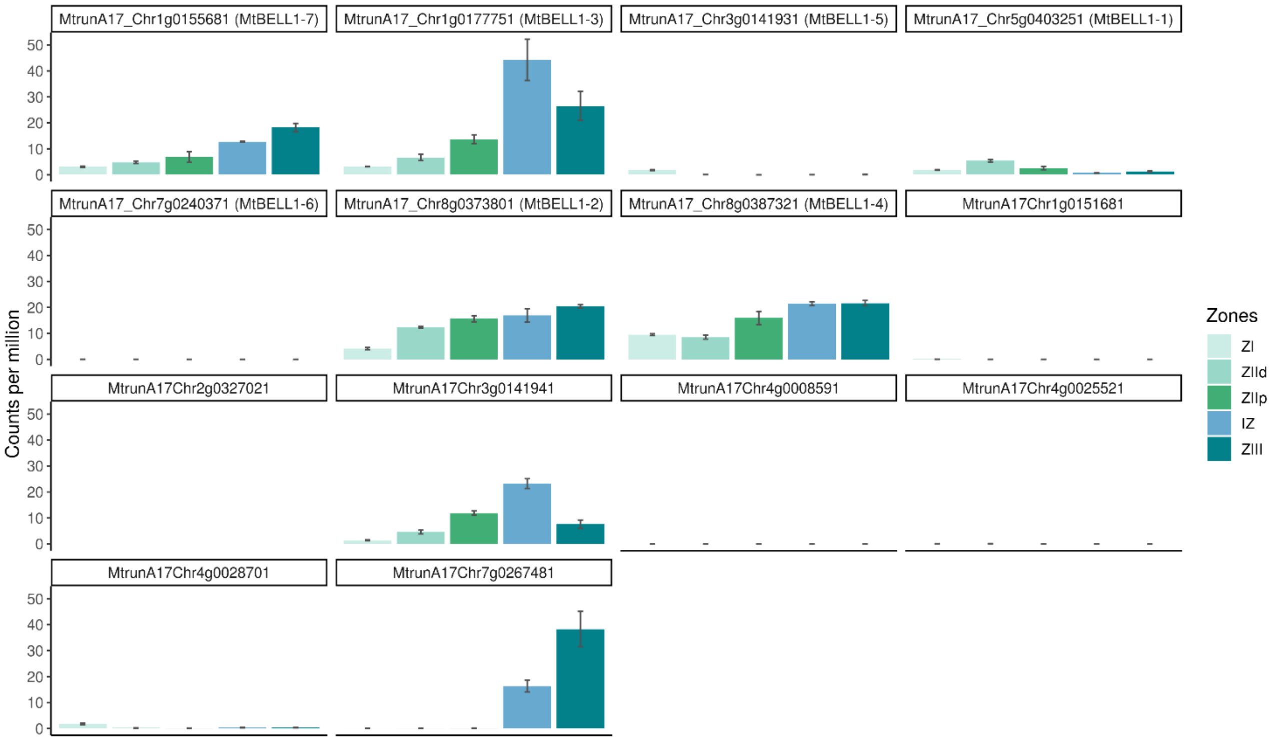

Figure S5. Expression level of *M. truncatula BELL* genes in the different nodule zones. Zone I (ZI) - bacteria free meristematic region; zone II (ZIIp) — the proximal part of the infection zone; zone II (ZIId) — the distal part of the infection zone; zone III (ZIII) — symbiosomes, which consist of differentiated bacteroids; the interzone (IZ) - a few cell layers wide between ZII and ZIII.

Table S3. List of primers used in this study.

RT-PCR primers:	
PsBELL1-1_F	TCAACAGCAGCAACAACAACAAAC
PsBELL1-1_R	AGAGGGAGGGCTAGGGAGTAACAT
PsBELL1-2_F	CTCACGGCGCCTCTCCTG
PsBELL1-2_R	TGAAATATGCTGCTGCTGCTACTG
PsBELL1-3_F	GGTTCTGTTGGTGGTGGTGAT GG
PsBELL1-3_R	TTGGCCCGTGATTGCGTC TT
PsBELL1-4_F	TTTCTCCATCCGTATCCAAG
PsBELL1-4_R	ACCAATTTGATACCTGGC
PsBELL1-5_F	GGAGACCCATTCGAGGCTTACC
PsBELL1-5_R	GCTGCTGGATTGGAGTCTTCTGAT
PsBELL1-6_F	ACTTTCTCCACCCTTATCCTACTG
PsBELL1-6_R	TTCTCGGCAATGTTGTTATCAC
PsBELL1-7_F	TTCGCTAAGTCTTGCAACATCTCC
PsBELL1-7_R	TATTTCCTTGCCCTCTAACACCAG
Primers for CDS cloning in pDONR221 or pEntry-TOPO vectors	
*PsDELLA1*_BP_F	AAAGCAGGCTTCATGAAGAGAGATCGTCAAGAAACC
*PsDELLA1*_BP_R	AAGCTGGGTGTCACTTTGACTCACTGAGTGGAA
*PsKNOX9*_BP_F	AAAGCAGGCTTCATGGCTTTTCACGACCATCT
*PsKNOX9*_BP_R	AAGCTGGGTGTCACATGAAGCTCTGATT
*PsBELL1-2*_BP_F	CACCATGGGGATAGCAACAACT
*PsBELL1-2*_BP_R	TTAAATGCCTCCAAAGTCTGTAAG
